# Supplementary material for: Predictors of Influenza Vaccination Uptake and the Role of Health Literacy among Health and Social Care Volunteers in the Province of Prato (Italy)
Source: Int J Environ Res Public Health. 2022 May 30;19(11):6688. doi: 10.3390/ijerph19116688 (PMC9180793; doi:10.3390/ijerph19116688)
Supplement: Supplementary file 1 [file ijerph-19-06688-s001.zip › ijerph-1690071-supplementary.pdf]

SUPPLEMENTARY MATERIALS

File S1: Questionnaire

SOCIODEMOGRAPHIC DATA

Date of the enrollment: dd/mm/yy

Sex: Female []; male []

Birth date: dd/mm/yy

Nationality: Italian []; Other (specify): _________

Educational level (choose the highest obtained): Primary school or less []; Lower secondary school []; High school []; Bachelor or higher []

Do you have any other job in addition to those in the voluntary association/institution? Yes []; no []

If yes, what kind of job is it? Health worker []; social-health worker [], Public employement [], non-public employment [],

HEALTH LITERACY

Ranging from very easy to very difficult, in your opinion, how much is difficult to:

- Understand the information disseminated by media (such as radio, television, newspapers, and internet) about how to improve your health? Very difficult []; quite difficult []; I do not know; [] quite easy []; very easy []
- Assess whether information about health risks referred by media is reliable? Very difficult []; quite difficult []; I do not know; [] quite easy []; very easy []
- Finding information on how manage health problems such as stress or depression? Very difficult []; quite difficult []; I do not know; [] quite easy []; very easy []
- Identify activities which are good for mental health? Very difficult []; quite difficult []; I do not know; [] quite easy []; very easy []
- Use the information referred by the physician to decide about your disease condition? Very difficult []; quite difficult []; I do not know; [] quite easy []; very easy []
- Evaluate when you need to get a second opinion by another physician? Very difficult []; quite difficult []; I do not know; [] quite easy []; very easy []

LIVING CONDITION

Number of cohabitants (including himself/herself):

Number of rooms in the house (excluding bathrooms and secondary rooms)

Among your cohabitants, is there people aged >64 years old and/or affected by chronic diseases or immunodeficiency? yes []; no []

RISK CONDITIONS OR DISEASES

Do you have one or more of the following risk conditions or diseases?

- Diabetes: yes []; no [], I do not know/I do not remember []

- Obesity: yes []; no [], I do not know/I do not remember []
- heart disease (for example ischemic heart disease): yes []; no [], I do not know/I do not remember []
- pulmonary diseases (for example asthma, chronic obstructive pulmonary disease): yes []; no [], I do not know/I do not remember []
- diseases of the immune system: yes []; no [], I do not know/I do not remember []
- chronic kidney diseases: yes []; no [], I do not know/I do not remember []
- chronic liver disease: yes []; no [], I do not know/I do not remember []
- organ or bone marrow transplant: yes []; no [], I do not know/I do not remember []
- chronic neurological diseases: yes []; no [], I do not know/I do not remember []
- oncological diseases (last 5 years): yes []; no [], I do not know/I do not remember []
- hematological diseases: yes []; no [], I do not know/I do not remember []
- pregnancy: yes []; no [], I do not know/I do not remember []
- surgery under general anesthesia (last year): yes []; no [], I do not know/I do not remember []

SMOKING HABITS

Do you smoke? I have never smoke (less than 100 cigarettes during your life) []; I am a former smoker []; Yes, less than 10 cigarettes/die []; Yes, 10-20 cigarettes/die []; Yes, more than20 cigarettes/die []

FLU VACCINATION

Did you get the flu vaccination last fall? yes []; no [], I do not know/I do not remember []
